# Effect of Vestibular Stimulation on Balance and Gait in Parkinson’s Disease: A Systematic Review

**DOI:** 10.3390/jfmk9040206

**Published:** 2024-10-25

**Authors:** Ardavan Iravani-Naeeni, Amir Mohagheghi

**Affiliations:** Centre for Cognitive and Clinical Neuroscience, College of Health, Medicine and Life Sciences, Brunel University London, London UB8 3PH, UK; ardavan.iravaninaeeni@brunel.ac.uk

**Keywords:** Parkinson’s disease, vestibular stimulation, balance, gait, ROBINS-I

## Abstract

**Background/Objectives:** Parkinson’s Disease (PD) can be associated with balance and gait impairments leading to increased risk of falls. Several studies have reported positive effects of various forms of vestibular stimulation (VS) for improving balance and stability in people with PD (PwP). The purpose of present study was to synthesise the current evidence on the effectiveness of VS, highlighting its potential benefits in improving postural stability and reducing gait impairments in people with Parkinson’s Disease. **Method:** A systematic search was conducted across databases Cochrane, Medline, PEDro, PubMed, Web of Science, and Google Scholar. Studies were included if they involved PwP at stages 3 or 4 of the Hoehn and Yahr scale, aged 60 years or older. The Risk of Bias (RoB) was assessed using the ROBINS-I tool. The review followed the PRISMA guidelines and the protocol was registered with PROSPERO (CRD42022283898). **Results:** demonstrated that various forms of VS have shown promise in mitigating symptoms of vestibular dysfunction and improving gait and balance in PwP. However, the overall RoB ranged from moderate to critical, with variations across different domains. **Conclusions:** While VS appears to offer potential benefits in improving balance and gait in PwP, the presence of biases in the reviewed studies necessitate caution in interpreting the results. Further research should focus on addressing these biases to confirm the therapeutic potential of VS in PD.

## 1. Introduction

Parkinson’s Disease (PD) is mainly diagnosed based on specific motor symptoms: tremor, rigidity, slowness of movement, and postural instability [1]. Among these symptoms postural instability present significant challenges for People with Parkinson (PwP), primarily due to impaired mechanisms for maintaining posture [2], but patients do not usually experience balance-related symptoms earlier than stage 3 according to H&Y scale [3]. Varied pathophysiological mechanisms for postural instability in PwP are proposed. One such factor is the deficits of the (peripheral and/or central) vestibular system.

Pastor et al. investigated the role of vestibular dysfunction as a primary cause of postural instability in PD and reported no significant differences between patients and healthy controls in body sway responses induced by galvanic vestibular stimulation [4]. However, since then several studies have suggested vestibular deficits in PwP and linked these with balance dysfunction. For instance, Schindlbeck et al. reported that PwP who had balance impairments also had a relative inability to perceive vertical position [5], suggesting impairment of the vestibular system at both the peripheral and central levels. Bertolini et al. reported that a vestibular deficit, likely attributed to higher-order sensory integration centers including the basal ganglia, was present in PD patients [6]. Pollak et al. [7] reported significantly higher number of abnormal Vestibular Evoked Myogenic Potentials (VEMP) responses in PwP compared to the control group. The study concluded that an abnormal vestibulocollic reflex (VCR) along with an impaired vestibulo-ocular reflex (VOR) might contribute to postural instability in PD patients. Reichert et al. [8] reported absent caloric responses from the vestibular system in PwP, suggesting that impaired peripheral vestibular function could contribute to postural instability and balance.

Evidence for the effect of pharmacological interventions on postural instability and gait in PD is limited. Rocchi et al. concluded that abnormalities in postural sway in patients with Parkinson who took levodopa increased and there was no significant improvement in balance with levodopa treatment [9]. They suggested that these findings might be the result of the effect of levodopa on reducing stiffness without improving postural control. Curtze et al. reported very small gait improvement with levodopa, but worsening of balance and increase in the risk of falls. They concluded that manipulation of the neurotransmitters in the cortical and brainstem circuits rather than dopamine replacement therapy may be needed to improve the condition [10].

Different approaches to improving balance may be adopted based on the underlying pathophysiological mechanism [11,12]. There is evidence for the potential benefit of stimulating the vestibular nerve via Galvanic Vestibular Stimulation (GVS) or vestibular receptors via temperature-induced movement of the endolymph through Caloric Vestibular Stimulation (CVS), which require minimal active participation from the patients, on balance and gait in PD [13]. Possible mechanisms for the effect of therapeutic vestibular stimulation using GVS and CVS for improving balance and locomotion may include enhancing neural plasticity in other brain structures that receive vestibular input. This may help with the central processing of the vestibular information in the process of sensory reweighting, or may enhance sensory sensitivity to the incoming vestibular afferents in basal ganglia [14]. Therefore, the remaining striatal cells may work more efficiently which consequently improve balance and coordination of movement. Pires et al. [15] also found evidence for the potential benefit of direct stimulation of the vestibular system to improve gait and reduce risk of fall in patients with PD. They attributed this effect to the enhanced integration of vestibular, visual and proprioceptive inputs and neuroplastic changes in the brain, helping to rewire neural pathways involved in the control of balance and movement.

Beneficial effects of vestibular stimulation (VS) on balance and gait are consistently reported, but there is an argument that small sample size, methods of presentation of data, inconsistent protocols for the effective use of vestibular stimulation, and baseline differences between the control and the experimental groups may have affected reported results.

The aim of this systematic review was to examine how well vestibular stimulation (VS) can improve balance, postural stability and gait in PwP who were at later stages of the disease according to Hoehn and Yahr (H&Y) scale by reviewing the effect of GVS, CVS and exercises (including vestibular rehabilitation techniques) that naturally stimulate vestibular system (NVS) on these measures.

## 2. Materials and Methods

The protocol of the study was registered with PROSPERO (CRD42022283898). Scope of the review was decided following a PICO model, and PRISMA guidance and checklist (PRISMA 2020) were used for reporting relevant items of the methodology.

### 2.1. Criteria for Inclusion of Studies

#### 2.1.1. Population

Studies were eligible for inclusion if they involved people with PD at stages 3 or 4 of H&Y scale, and aged 60 years or older. Reasons for these criteria were that only 4% of people with PD are below 50 years of age and the incidence of PD rapidly increases over the age of 60 years [16], and that loss of balance and slowness of movements appear at stage 3 on H&Y scale.

#### 2.1.2. Experimental Intervention/Training

Studies that tested the effects of the following various forms of VS were included: Galvanic Vestibular Stimulation, Caloric Vestibular Stimulation, and Natural Vestibular Stimulation (natural movements with a vestibular specific component).

#### 2.1.3. Outcome Measures

All studies that investigated the effect of VS on balance and gait outcomes were eligible for inclusion regardless of the presence of retention test. This included biomechanical outcome measures of (i) postural stability/balance related to the behavior of center of pressure (COP) and (ii) spatiotemporal characteristics of gait. Clinical measurements were limited to Berg Balance Score (BBS), Functional Reach Test (FRT), Timed-Up and Go (TUG), Four Square Step Test (FSST), Limits Of Stability (LOS), Biodex Balance System (Static and Dynamic), modified Clinical Test of Sensory Organisation on Balance (mCTSIB), Freezing of gait (FoG) and Anticipatory Postural Adjustment (APA) deficiency.

#### 2.1.4. Study Design

Experimental groups were compared against Control groups consisting of PwP who were “on” or “off” medication and received either no intervention (passive control) or alternative therapeutic interventions (active control).

### 2.2. Search Strategy and Study Selection

Cochrane, Medline, PEDro, PubMed, Web of Science and Google Scholar databases were searched to find relevant artciles. Four main keywords, i.e., Parkinson’s Disease, Vestibular Stimulation, Gait, and Balance were used to identify records within databases. Keywords of Parkinson’s Disease (Parkinson), vestibular stimulation, balance (balance, stability, postural stability, locomotion, instability, gait and fall) were also identified. Search strategies developed using those terms for each database are shown (see Table 1). Searches were restricted to English language and from 1971 through 8 May 2022. The final search syntax which were reviewed and agreed upon by two researchers (AI and AM) are included in Table 1.

Retrieved articles from all databases were merged, with duplicates removed. Two independent reviewers (AI and AM) conducted screening of Titles and Abstracts. Full text screening of potentially relevant articles was conducted by AI and checked by AM. The reference lists of eligible studies were hand searched to identify additional publications. Data extraction was conducted by (AI) and another reviewer (AM) checked all data entry. Corresponding authors of individual studies were contacted via email and additional data relevant to the present study inclusion criteria was requested if, for example, these studies included participants who were younger than 60 or had H&Y scores other than 3 or 4.

### 2.3. Synthesis and Analysis

The ROBINS-I tool [17] was used to assess Risk of Bias (RoB) and appraise articles strength and weaknesses. The tool evaluates seven domains of bias. The first two domains, are related to confounding parameters and selection of participants into the study and bias that might have happened before the start of the interventions. The third domain addresses classification of the interventions and the other four domains address bias introduced after the start of the intervention. RoB for each domain was classified as low, moderate, serious, critical, or having no information and the final overall evaluation for each study was based on the finding from each domain.

## 3. Results

**Figure 1:**
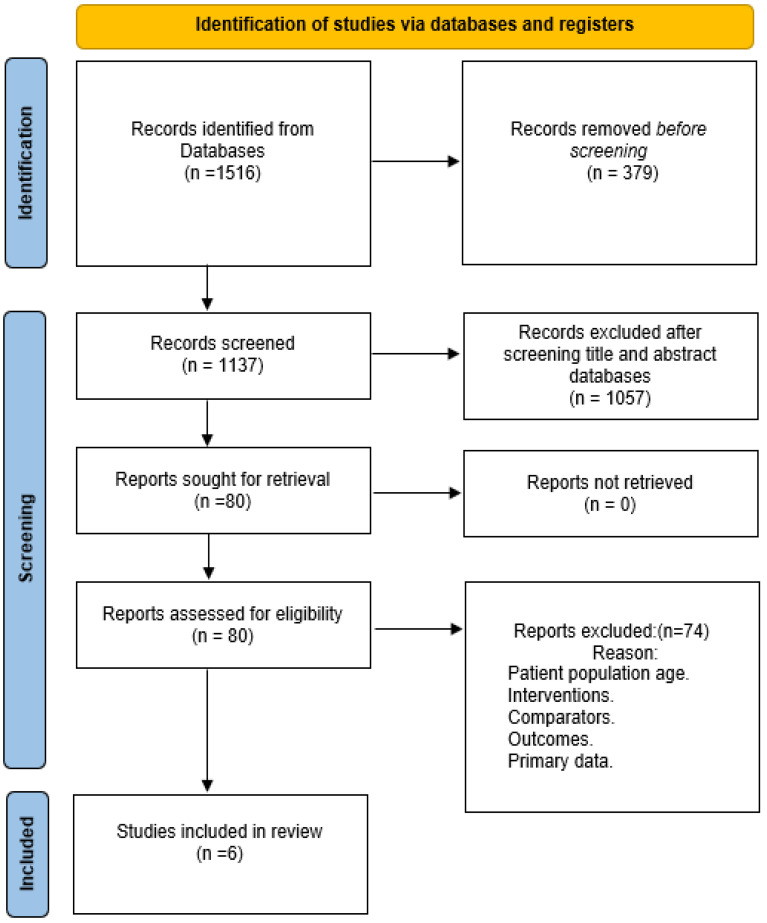
Process of identification of qualified studies.

Out of 1516 studies which were initially retrieved, 6 were qualified for inclusion in the review. Figure 1 illustrates process of identification of the qualified studies.

Table 2 includes detailed characteristics of the qualified studies. Qualified studies demonstrated improvements in the selected outcome measures following vestibular stimulation employing a range of different interventions.

Reduction in TUG was reported by Wilkinson et al. [21] and Wilkinson et al. [22] who utilised different caloric vestibular stimulation devices. Rossi-Izquierdo et al. [24] applied rehabilitation exercises with computerised dynamic posturography (CDP) to induce vestibular stimulation. Fil-Balkan et al. [18] and Acarer et al. [20] interventions were purely exercise based, but similarly led to significant reduction in TUG with training.

Bonni et al. [23] reported an increased relative duration of the single-legged stance and double-support phases, as well as a reduced duration of the swing phase, after blindfolded balance training compared to routine physiotherapy. This was despite the latter intervention including balance training on an unstable platform and gait training.

Improved BBS scores were reported in the studies by Acarer et al. [20] and Fil-Balkan et al. [18]. Fil-Balkan et al. also noted improved BBS scores in their control group who only received classic physiotherapy for 6 weeks. However, the improvement in the study group, who received sensory motor integration training in addition to classic physiotherapy, was reported to be significantly higher both after the completion of the intervention (6 weeks) and at follow-up (12 weeks). Results for the FRT were similar.

Only one study [22] reported the results of vestibular rehabilitation on Limits of Stability using CDP. The difficulty of training was customised and increased throughout the program, resulting in a significant increase in all LOS related outcome measures after training, which persisted for at least one year.

Changes in the mCTSIB following customised rehabilitation intervention was reported by Acarer et al. [20]. Data reported in their Table 3 (p. 259) suggests decreased sway speed in the rehabilitation group for the eyes open condition on both firm and foam surfaces, indicative of improvement in balance abilities.

Protocol consideration for the “Target trial” against which assessment of RoB was completed is presented in Table 3.

**Figure 2:**
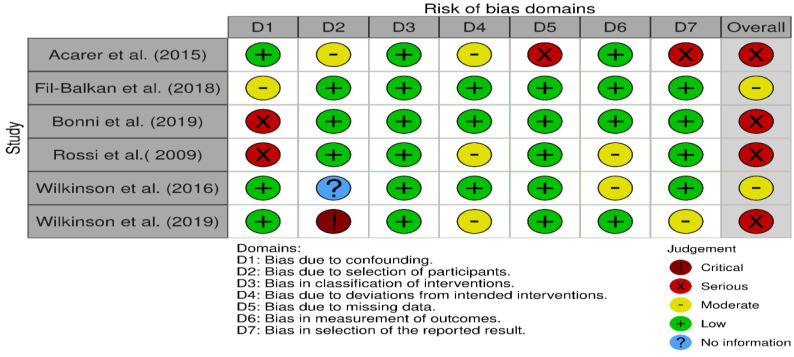
Risk of Bias in different domains in the qualified studies [18,20,21,22,23,24].

**Figure 3:**
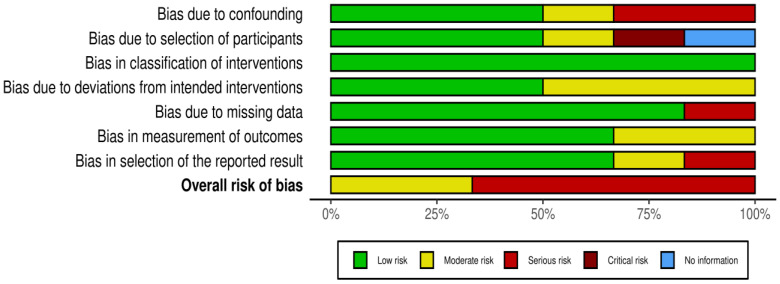
Graphical summary of the overall RoB.

Figure 2 shows ROB in different domains across qualified studies, and Figure 3 is a graphical summary of the overall assessment of ROB. Overall and across all domains, 4 studies had serious RoB, and 2 had a moderate RoB.

## 4. Discussion

This systematic review examined evidence from studies which investigated the effectiveness of different forms of Vestibular Simulation interventions on selected measures of balance and gait in patients with Parkinson’s Disease. Interventions for stimulating vestibular system, which included purposeful movements and exercises (collectively examined under natural vestibular stimulation), or stimulation using external devices (Galvanic and Caloric Vestibular Stimulation) were reported to positively affect different aspects of walking and postural abilities in PwP. Possible mechanisms for the effect of therapeutic vestibular stimulation using GVS and CVS for improving balance and locomotion may include enhancing neural plasticity in other brain structures, to where direct vestibular inputs project. This may help with the central processing of the vestibular information in the process of sensory reweighting [25]. Furthermore, vestibular stimulation may enhance sensory sensitivity to the incoming vestibular afferents in basal ganglia [26]. Therefore, the remaining cells can work with more efficiency which may consequently improve balance and coordination of movement. Consistency of the reported findings, regardless of the methodology employed, suggest that vestibular stimulation can be an effective intervention for enhancing balance and postural steadiness in PD, and it had a positive effect on motor performance after being delivered for minimum two weeks and frequency of 10 sessions for minimum 20 min.

There were methodological issues concerning statistical approaches to analysing the results. Not all studies [18] analysed differences between an experimental and control group at baseline with differences after completion of the study and/or follow-up [27] excluded.

The practice can potentially ignore natural changes over time and regression towards the mean in the outcome measures.

Despite reported overall positive findings, we argue that the presence of biases in the qualified studies, might have also contributed to the observed outcomes. We identified biases in all domains, apart from Domain 3 (bias in the classification of interventions). Addressing causes of bias will be necessary in any future study designs which investigate effect of vestibular stimulation on improving balance or gait.

Domain 5 (bias due to missing data), placed one of the qualified studies [20] at serious RoB. Proportions of participants for whom data was available for the analysis differed substantially across intervention (n = 29) and control groups (n = 11) in the Acarer et al. study. In Fil-Balkan et al. study [18], from patients who started the study (n = 34), only 44% (n = 15) were assessed at the end of follow-up period. Although reasons for patients drop out were not reported, there is a possibility that interventions had adverse effects or were intolerable to participants. Particularly, lost to follow up was more noticeable in studies where participants had to commit to physical activities such as those in Acarer et al. and Fil-Balkan et al. [18,20].

Participants’ general characteristics should be assessed within every study because of their association with balance impairment and falls. For example, effects of high Blood Pressure, Arthritis, Obesity, Vitamin B-12 Deficiency, Stroke, Migraine, Head injury, Diabetes, and any medications for pain should be accounted in all studies related to the assessment of balance and risk of fall as they may confound variables being assessed or show significant differences between (experimental and control) groups at baseline. Three of the reviewed studies [18,23,24] did not consider one or more of these characteristics and were considered as having moderate or severe RoB according to Domain 1 (bias due to confounding) in ROBINS-I. As this domain may affect both interventions and outcomes of interest, we suggest researchers to recognise all known confounding variables, report any differences between groups and control/adjust for these variables, where possible. We have suggested a protocol of considerations for Target Trial that may be adopted and used to this end (Table 3).

Three studies [20,22,24] had moderate RoB (Figure 2) due to departures from intended interventions (Domain 4). In Acarer et al. interventions were intentionally personalised and it may be argued that did not introduce bias in outcomes. However, in the design of observational studies, it is ideal to standardise intervention protocols and avoid co-interventions.

With regard to bias in the measurement of outcomes (Domain 6), majority of studies (4 out of 6) were classified as having low RoB (Figure 2), with only 2 considered at moderate level. Rossi- Izquierdo et al. study was considered as moderate for this domain, because the assessor could not be blinded. Ideally for this domain, assessors should be blinded to interventions status of collected data.

Domain 7 (Bias in selection of the reported results) was difficult to assess for two studies [20,22] as authors did not fully report their protocols.

Finally, bias due to selection of participants into the study (Domain 2) in the overall assessment of RoB, was judged at serious based on one [22] out of 6 studies. The bias could have arisen due to inclusion of prevalent users and/or not including results of all participants after initiation of the intervention.

## 5. Conclusions

To conclude, this review suggests potential positive effects on aspects of balance and gait by vestibular stimulation in patients with Parkinson’s Disease, but these results should be interpreted with caution. It is evident from the limited number of studies qualified for this review (six) that published data on this topic is extremely scarce. Importantly, these studies employed various interventions for vestibular stimulation, making it difficult to reach firm conclusions about the best therapeutic approach, identify potential “best” responders to vestibular stimulation, or understand the underlying mechanisms of action. Although the results suggest improvements in gait, gait control, balance, postural control, and mobility [18,20,21,22,23,24], drawing definitive conclusions remains challenging. Consequently, we argue that there is a need for further high-quality research to better understand the effectiveness of vestibular stimulation for improving balance and gait, and specific conditions under which and populations in whom vestibular-based interventions are most effective. Long-term sustainability of the observed benefits should also be examined. Following guidelines of ROBINS-I tool, and recommended protocol for creating a target trial [17], the proposed target trial (Table 3) can be considered for designing studies in which the effect of vestibular stimulation for improving aspects of gait and locomotion are examined.

As our understanding of the vestibular system and its interactions with other sensory systems expands, new opportunities for use of vestibular stimulation emerge. Researchers are currently exploring its potential in areas such as sports performance enhancement [28], motion sickness prevention [29], cognitive rehabilitation [30], and treatment of mental health [31]. This systematic review provides evidence supporting the effectiveness of vestibular stimulation interventions in reducing risk of falls by affecting measures which indirectly show improvement in balance and walking abilities, although RoB in different domains were present and should be considered alongside reported results. Vestibular stimulation intervention offers a promising approach to traditional therapeutic modalities and may be considered as an addition to treatment plans in clinical and community settings. Future research should focus on strengthening the evidence and optimising protocols for the implementation of vestibular stimulation as part of patients’ therapeutic interventions.

## Figures and Tables

**Table 1:** Search syntax employed for different databases.

Database	Syntax
PubMed	(“Parkinson Disease”[Mesh] OR Parkinson* OR parkinson’s disease OR parkinson disease OR parkinsons disease OR parkinsons OR parkinsonism) AND (“Vestibular System”[Mesh] OR “Vestibular Stimul*”) AND (Balance OR Stability OR “postural stability” OR instability OR gait OR fall OR “Postural Balance”[Mesh] OR “Gait”[Mesh] OR walking OR locomotion OR mobility OR posture OR posture control OR postural sway)
EbscoHost Search (Academic Search Complete, Medline, CINAHL)	(Parkinson* OR parkinson’s disease OR parkinson disease OR parkinsons disease OR parkinsons OR parkinsonism) AND (vestibular N3 stimulat* OR “Vestibular System”) AND (gait OR walking OR locomotion OR mobility OR posture OR postural control OR postural stability OR postural sway OR balance OR instability OR fall)
Web of Science	(Parkinson* OR parkinson’s disease OR parkinson disease OR parkinsons disease OR parkinsons OR parkinsonism) AND (vestibular stimulat* OR “Vestibular System”) AND (gait OR walking OR locomotion OR mobility OR posture OR postural control OR postural stability OR postural sway OR balance OR instability OR fall)
Google Scholar	Parkinson balance OR fall OR locomotion OR stability OR instability “vestibular stimulation”
Cochrance:	1–((Parkinson* OR parkinson’s disease OR parkinson disease OR parkinsons disease OR parkinsons OR parkinsonism)):ti,ab,kw 2–((vestibular stimulat* OR “Vestibular System”)):ti,ab,kw 3–((gait OR walking OR locomotion OR mobility OR posture OR postural control OR postural stability OR postural sway OR balance OR instability OR fall)):ti,ab,kw #1 AND #2 AND #3
PEDro	(Parkinson*balance) (Parkinson*gait) (Parkinson*fall) (Parkinson*locomotion) (Parkinson*stability) (Parkinson*instability)

**Table 2:** Study characteristics of the qualified studies.

Authors	Participants	Interventions	Outcome Measure	Notes
Fil-Balkan et al. (2018) [18]	34 participants assessed for eligibility. 30 participants allocated to Study n = 15 Control groups n = 15. 26 participants completed 6-week training: Study group n = 12. Control group n = 14. 24 participants continued to complete and reassessed at the 7th week: Study group n = 12; Control group n = 12. 15 participants completed to follow-up and reassessed at the 12th week: Study group n = 7; Control group n = 8. Inclusion criteria: Idiopathic PD Age: ≥50 years Modified H&Y score: 2–3 (2.5/3) Mini Mental Test score: ≥26 No changes to medications (Levodopa, Cardbidopa, dopamine agonists) during the study. Exclusion criteria: Severe mental or psychological disorder Physiotherapy for the last 6 months. Study group BBS: Mean 40.91 ± 7.91 Control group BBS: Mean 42.17 ± 12.26 *p* = 0.488 (Mann Whitney U test) Study group TUG: Mean 16.59 ± 8.29 Control group TUG: Mean 16.75 ± 10.45 *p* = 0.583 (Mann Whitney U test)	Control group: Twice a week 1-h classic physiotherapy for 6 weeks. Classic physiotherapy training included: person-specific flexibility, strengthening, posture, breathing, balance according to Cattaneo et al. (2007) [19], walking exercises, and other functional activities (not defined specifically) intensified with the progress of an individual’s performance, tolerance and needs. Study group: Classic physiotherapy as above + 30 min Sensory Motor Integration Training (SMIT) during each session. SMIT training included: Proprioceptive, visual and vestibular stimulation. A sensorimotor-perceptual integration activity was also designed and used in the form of a “walking trail” to improve motor control components of postural control.	Measurements were made at: Baseline: One week before the intervention. Week 7: During the first week right after the intervention. Follow-up: Week 12 (6 weeks after the last intervention session). Clinical Measurements: TUG: Mean of 3 attempts. FRT BBS Statistical analysis: Non-parametric analysis (Wilcoxon Signed Rank Test for intragroup comparisons; Mann-Whitney U test for inter-group comparisons) of change in score (Δscore) between pre-therapy and post-therapy (T1–T0), and pre-therapy and follow-up (T2–T0) was used. Cohen’s d Effect size was calculated. CI was calculated for postural control values. α = 0.05 Results: Study group BBS: T1–T0 (Δweek7&0): 10.42 ± 8.48 (Mean ± SD) 95% CI: [5.03–15.80] Significant improvement. T2–T0 (Δweek12&0): 11.71 ± 10.90 (Mean ± SD); 95% CI: [1.63–21.80] Significant improvement. Control group BBS: T1–T0 (Δweek7&0): 4.25 ± 4.31 (Mean ± SD) CI: [1.5–6.99] Significant improvement. T2–T0 (Δweek12&0): 1.37 ± 8.79 (Mean ± SD) CI: [−5.98–8.73] Non-significant improvement. Patients in the Study group showed more improvements compared to those in the Control group (*p* = 0.027) post treatment and at follow-up (*p* = 0.037). Study group TUG: T1–T0 (Δweek7&0): −4.74 ± 2.01 (Mean ± SD) CI: [−6.02–−3.47] Significant improvement. T2–T0 (Δweek12&0): −4.80 ± 2.34 (Mean ± SD); CI: [−6.97–−2.64] Significant improvement. Control group TUG: T1–T0 (Δweek7&0): −2.36 ± 2.17 (Mean ± SD) CI: [−3.75–−0.98] Significant improvement. T2–T0 (Δweek12&0): −0.69 ± 1.21 (Mean ± SD) CI: [−1.71–0.32] Non-significant improvement. Patients in the Study group showed more improvements compared to those in the Control group (*p* = 0.001) post treatment and at follow-up (*p* = 0.002). Study group FRT: T1–T0 (Δweek7&0): 6.43 ± 3.00 (Mean ± SD) CI: [4.53–8.34] Significant improvement. T2–T0 (Δweek12&0): 6.14 ± 4.56 (Mean ± SD); CI: [1.92–10.36] Significant improvement. Control group FRT: T1–T0 (Δweek7&0): 3.52 ± 2.59 (Mean ± SD) CI: [1.87–5.16] Significant improvement. T2–T0 (Δweek12&0): 2.62 ± 3.34 (Mean ± SD) CI: [−0.17–5.42] Non-significant improvement. Patients in the Study group showed more improvements compared to those in the Control group (*p* = 0.024) post treatment but not at follow up (*p* = 0.115).	Design: Randomised Controlled Trial Evaluations completed during the “ON” period. Study and Control groups were different at the start of the study for SOT 6th position with the participants in the Control group scoring higher. (Mann Whitney U test) Improvements in the clinical measures after intervention for the Study group. For the Control group, TUG reduced after 6 weeks, too. There is also improvement in vestibular and visual system score for both Study and Control groups (but not supported by the reported values in Table 3 for vestibular system score). 6 weeks follow up assessments shows that improvement was maintained in the Study group even after 6 weeks compared with control group and authors suggested that this positive effect was due to SMIT. Authors contributed improvement in postural control to increasing capacity of the vestibular system.
Acarer et al. (2015) [20]	60 participants were randomized into: Treatment/Group 1, n = 30; Control/Group 2, n = 30. 40 participants completed the study: Rehabilitation Group: n = 29 completed n = 1 dropped due to difficulty in commuting Age (mean (range)): 67 (51–81) years Sex (M/F): 17/12 Length of illness (mean (range)): 4.5 (1–24) years History of falling: 13 (44.8%) H&Y score: phase II: 22 (75%) phase III: 7 (25%) Control Group: n = 11 completed n = 19 dropped out due to change in medication (6), withdrawal (lost to follow-up; 11), unwilling to complete the final assessments (2). Age (mean (range)): 60 (40–71) years Sex (M/F): 8/3 Length of illness (mean (range)): 8 (1–18) years History of falling: 8 (72.7%) H&Y score: phase II: 6 (54.5%) phase III: 5 (45.5%) Participants had: no history of peripheral vestibular disease Normal oto-neurological examination Normal visual acuity Normal sense of position and vibration Inclusion criteria: Idiopathic PD Age: ≥40 years H&Y score: II (n = 28); III (n = 12) Adapted to medication and able to walk indoor independently without walking aid. Exclusion criteria: Conditions limiting therapeutic exercises (ambulatory problems, <30° cervical movement having no physiotherapy for the last 6 months Vestibular or visual or somatosensorial disorders Cognitive, orthopedic or other neurological problems. Mini-Mental test ≤ 24 Medical conditions affecting balance and gait, or participation in training	Rehabilitation group: Intervention exercises (hospital and home exercise programs): Hospital exercise program: Once per week for 8 weeks at hospital including: Adaptation exercises (one minute each condition, 3 times a day): Moving the head in a yaw rotation while maintaining gaze on a target in 2 conditions: (A) head rotated while focusing on stationary target; (B) Head and the target both move in opposite direction. Substitution exercises: Physiotherapist trained the patients to substitute a sensory system with the one with lost or poor function. Habituation exercises: Walking with turning the head side to side. Balance exercises: Restoring balance while moving from a static (e.g., standing) position to another dynamic (e.g., walking) position Home exercise program: Twice per day Selected from 4–5 exercises performed at hospital Control group: no exercises; received usual care.	Measurements were made at: Baseline: Before rehabilitation Week 8: After the intervention Measurements: mCTSIB TUG BBS Rehabilitation group BBS Before Rehab: 48 (8–56); After Rehab: 53 (21–56); *p* ˂ 0.05 Control group BBS: Before Rehab: 47 (29–52) After rehab: 44 (7–55); NS Rehabilitation group TUG: Before rehab: 12.2 (9–22) After Rehab: 10 (7–14); *p ˂* 0.05 Control group TUG: Before Rehab: 11.2 (77–22) After Rehab: 11.0 (9–14); NS mCTSIB: FIRM EO (After rehab): Rehabilitation group: 0.33 [0.1–0.8] Control group: 0.40 [0.2–1.3] *p* < 0.05 FOAM EO (After rehab): Rehabilitation group: 0.72 [0.3–1.3] Control group: 0.8 [0.4–2.2] *p* < 0.05 FIRM EC (After rehab): Rehabilitation group: 0.3 [0.1–1.13] Control group: 0.4 [0.1–1.7] *p* > 0.05 FOAM EC (After rehab): Rehabilitation group: 1 [0.6–2.6] Control group: 1.6 [0.7–2.5] *p* > 0.05	Data in Table 1, Table 2 and Table 3 is poorly reported and it is not clear what they represent. Data in the current table is just a replication of what has been reported by authors. Participants remained on stable dose of PD medication 1 month before and throughout the study. Tests were completed 2 h after receiving medication in “ON” state. Improvement after 8 weeks vestibular rehabilitation on ABC, BBS, DGI, TUG. No improvement on UPDRS-m. participants have reported that their level of confidence improved but fear of fall was reduced. Authors stated an improvement in patients static posturography scores in Control group which was attributed to getting familiar with the test.
Wilkinson et al. (2016) [21]	Participant: n = 1 Sex: Male Age: 70 years H&Y Score:4 reported by direct contact with author Length of illness: 7 years symptoms: Hypokinesia, rigidity and memory lost symptoms. Medication: Stalevo 150 mg/25 mg/200 mg Levodopa 100 mg Carbidopa 25 mg Entacapone 200 mg Pramipexole dihydrochlorine 1 mg Remaining unchanged through the study.	Caloric Vestibular stimulator device (Scion Neurostim). One earpiece stimulated ear canal by a cold sawtooth waveform to 17 C every 2 min and the other earpiece stimulated ear canal to 42 C every 1 min and was switched every two days. 5 days a week for 3 months (1-month sham, 2 months active stimulation) Sessions ran for 20 min twice a day, min 4 h gap. CVS delivered with participant inclined in supine position and head flexed at 30 degrees.	Measurements were made at: Baseline: Two weeks before sham stimulation. End of Week 4 after sham stimulation. End of first and second months (Active stimulation) Follow-up: 5 months after the last stimulation. Outcome measures: TUG Two minute walk Results: TUG: Significant MCID between sham CVS scores (20.4 s at baseline) and after Active CVS (second month: 16.2 s) and Follow-up (13.3 s) Significant MCID between end of first (21.5 s) and second (16.2 s) month of Active CVS NS MCID between second month of Active CVS and Follow-up Two-minute walk: Significant MCID between second month of Active CVS/Follow up and other time points: Before Rehab 22 m; After sham stimulation: 36 m; After first month of Active CVS: 84 m; After Active second month: 120 m; Follow up: 102 m	The greatest improvement was observed after second month. High improvement reported for Mobility and cognition. Patient reported better sleep and less anxious.
Wilkinson et al. (2019) [22]	59 participants screened, 46 recruited. Treatment or active group, n = 23 Control or placebo group, n = 23 33 participants completed treatment period (week 12). Treatment Group: n = 16 Placebo Group: n = 17 31 participants completed follow up period (week 17). Treatment group n = 14 Placebo group n = 17 Treatment group n = 23 Age (range): 69.7 (SD 11.3) Sex (M): 12 (52.2%) Length of illness (years and range): 11 (2–28) H&Y score: 2.5 (1.5–4) Placebo Group: n = 23 Age (range): 72.2 (SD 6.6) Sex (M): 18 (78.3%) Length of illness (years and range): 5 (2–14) H&Y score: 2.0 (1.5–4) Participants criteria: no history of peripheral vestibular disease receiving stable doses of dopaminergic drugs. No neurostimulation experience	8 weeks Caloric Vestibular Stimulation (CVS) at home. Twice daily for 19 min. (minimum 1-h gap) Device: ThermoNeuroModulation(TNM) Warm-tooth thermal (37C–42C) Cold-tooth thermal (37C–17C) Every 2 days warm and cold waveforms was switched between ears	Measurements were made at: Baseline: 4 weeks before randomised selection. and Just before randomised selection. Week 8 (Midway treatment) Week 12 (End of treatment) Week 17 (5 weeks after end of treatment). Measurements: TUG 2-min walk Result: (α = 0.05) TUG: Before Rehab: (time to complete) Active group: 14.1 (7.6, 59.1) Placebo Group: 11.4 (6.5, 213.0) *p =* 0.983 Week 12: Active group (Mean): −1.1 Placebo group (Mean): 0.0 Therapeutic Gains: −1.3 95% CI: −3.7 to 0.5 Week 17: Active group (Mean): −0.9 Placebo group (Mean): 0.0 Therapeutic Gains: −0.7 95% CI: −2.7 to 1.1 2-min walk (Distance in meters) Before Rehab: Active group: 73.2 (25.3) Placebo Group: 77.4 (33.1) *p* = 0.642 Week 12: Active group (Mean): 0.3 Placebo group (Mean): −1.7 Therapeutic Gains: 2.0 95% CI: −7.7 to 11.8 Week 17: Active group (Mean): 4.8 Placebo group (Mean): −3.8 Therapeutic Gains: 8.6 95% CI: 0.4 to 17.5	Assessments were on medication state. Research partly supported by Scion NeuroStim, LLC.
Bonni et al. (2019) [23]	16 right handed participants (M = 7 F = 9) were randomly assigned to 2 groups. Physiotherapy (PT) group (Control): N = 8: M4/F4 Age (Mean ± SD): 66.6 ±6.9 years Disease duration (Mean ± SD): 7 ± 3.1 years UPDRS score (Mean ± SD): 20 ± 6.8 Blindfolded Balance Training (BBT) group (Experimental): N = 8: M3/F5 Age (Mean ± SD): 71.8 ± 3.1 years Disease duration (Mean ± SD): 5.2 ± 3 years UPDRS score (Mean ± SD): 21 ± 8.5 Inclusion criteria: All treated with Levodopa (600 ± 250 mg), and receiving stable doses for at least 2 weeks before study and during the study. Exclusion criteria: Antidepressant 2 months before study History of pacemaker or brain stimulation History of epilepsy or dementia (Mini Mental status ˂ 24) Serious medical condition or neurological disease	10 sessions of treatment over 2 weeks: 5 days per week, for 40 min: PT group: conventional physiotherapy including muscle stretching, active and assisted limb mobilization, four limbs coordination exercises, balance training on instable platform and gait training. BBT group: BBT consisting of balance and walking exercises to stimulate dynamic postural control and improve balance reactions. BBT included (1) marching on the spot on a foam cushion blindfolded and turning 90 degrees clockwise every 1 min; and (2) treadmill training during which blindfolded participant walked for 4 min. Initial speed was 1 km/h and increased 0.5 km/h every minute up to 3 km/h.	ANOVA: Time (Pre vs Post) as within-subjects and Group (BBT vs PT) as between-subjects factors; α = 0.05 Stance phase for the more affected body side (MABS) reduced significantly: Time effect (F_(1,14)_ = 19.53; *p* = 0.0006); Group main effect (F_(1,14)_ = 2.67; *p* = incorrectly reported in the original article as 12; NS); Time × Group interaction (F_(1,14)_ = 12.92; *p* = 0.003); Post-hoc: significant reduction of stance phase percentage following BBT (*p* < 0.0004) Double Stance phase for MABS reduced significantly: Time effect (F_(1,14)_ = 10.43; *p* = 0.006); Group main effect (F_(1,14)_ = 2.08; *p* = 0.17); Time × Group interaction (F_(1,14)_ = 8.43; *p* = 0.01); Post-hoc: significant reduction following BBT (*p* < 0.0004) Swing phase for MABS increased significantly: Time effect (F_(1,14)_ = 10.15; *p* = 0.006); Group main effect (F_(1,14)_ = 7.84; *p* = 0.002); Time × Group interaction (F_(1,14)_ = 10.15; *p* = 0.006); Post-hoc: significant increase following BBT (*p* = 0.002) Gait speed for MABS increased significantly: Time effect (F_(1,14)_ = 4.33; *p* = 0.056);	Authors claimed: Visual deprivation and proprioceptive perturbation may be useful to improve gait and postural control mediated through involvement of the vestibular system. Suggested neural mechanism involved was possibly improved connectivity of the SMA-M1 circuits. Study was a Controlled Randomised Trial Testing session was completed 2 h after first morning drug administration (i.e., in ON condition).
Rossi-Izquierdo et al. (2009) [24]	45 participants who were recruited for a former study were screened, 17 were selected but 7 dropped due to reasons unrelated to the study (death, surgery, lack of transport). Treatment group n = 10 (n = 8 took part in the follow-up; the other 2 changed location and were not accessible) Age: 69.3 years (range 48–80 years) Sex: M = 5, F = 5 Length of illness: 7.15 (4–19) years H&Y score: 6 patients at stage III; 4 patients at stage IV There was no Control Group, but measures of TUG from 20 healthy subjects who were age- and sex-matched was used to determine eligible Treatment group participants. Participants’ characteristics: TUG ˃ 15.90 s No dementia, autonomic disorders, postural instability or hallucinations. No other neurological, cochleovestibular or middle-ear alterations. No wheelchair users Continued their usual medication.	Vestibular rehabilitation via CDP: 9 sessions, half an hour, over 1 month 10 CDP exercises were delivered which were customised according to participant’s deficits. Difficulty of exercises increased throughout training by increasing LOS, transition rate or movement of the posturography platform. Participants took usual medication and were tested and trained in the “on” state.	Measurements: TUG (time, steps, supports, falls, value assigned to the performance of the test) LOS (reaction time, directional control, movement velocity and distance covered (endpoint and maximum excursion) within stability limits) Statistical test: comparing outcome measures before and after training and at 1-year follow-up using Wilcoxon test. Results: TUG: 22.90 s (SD 6.22; range 16–33 s) before vs 16.00 s (SD 6.28; range 11–33 s) after treatment *p* = 0.004 21.63 s (SD 6.27; range 16–33 s) before vs 17.5 s (SD 4.2; range 14–26 s) at 1-year follow-up *p* ˂ 0.006 Non-significant difference for: steps, value, support LOS: Significant improvement in reaction time (RT—s), lateral plane movement velocity (MVL—deg/s), lateral plane directional control (DCL—%), and endpoint & maximum excursion after completion of the study and 1-yr follow-up: All *ps* < 0.05 Significant improvement in all subscales: For emotional *p* = 0.006 For functional *p* = 0.037 For physical *p* = 0.008	Authors’ conclusion: vestibular rehabilitation is effective for gait velocity, balance and the risk of fall (based on improvement in TUG). Benefits persist over time. Note: no control group Authors claimed rehabilitation can be customised for each individual.

**Table 3:** Protocol considerations for Target trial.

Eligibility Criteria	Parkinson’s Disease according to the clinical diagnostic criteria of the UK Parkinson’s Disease Society Brain Bank Age 60 to 65 3 to 7 years from time of diagnosis H&Y score ≥ 3 No history of FoG within the past 12 months Stable dose of PD medication at least 1 month before and during the intervention. Ability to ambulate indoor independently (nonwheelchair-bound). No relevant rehabilitations within the past 6 months. No other Neurological or musculoskeletal disorders. Peripheral vestibular system (PVS) specific: Normal heave (otolith function) and rotational (semicircular canals function) head impulses, no vertical ocular misalignment to suggest otolith imbalance No medical conditions affecting gait & Balance: Blood pressure, Arthritis, Obesity, Vitamin B-12 deficiency, Stroke, Migraine, Head injury, Diabetes, any medications for pain, depression, hallucination, autonomic disorder, cochleovestibular or middle ear alterations).
Treatment Strategies	Vestibular stimulation (VS) Physical therapy (PT) Behavioural therapy (BT) Taking VS, PT, BT at the baseline and remained on it during the follow up
Assignment procedures	Random allocation to a treatment strategy Blinded assessment
Outcomes	Postural stability & balance (TUG, 2-min walk distance, Time for 10-m walk, BBS, FOG) Gait Fatigue severity scale Function and Quality of Life
Follow up	Behaviour assessment (e.g., Baseline, Midway, at the end and 5 weeks after treatment) or loss to follow up or death, whichever occurs first Lag time for availability of administration records
Causal contrast of interest	Intention to treat effect, per-protocol effects using observational data (effect of Vestibular Stimulation on balance, stability, gait)
Statistical methods	Intention to treat analysis to compare the outcomes of the groups assigned to each treatment strategy.
